# Serum vitamin D status and circulating irisin levels in older adults with sarcopenia

**DOI:** 10.3389/fnut.2022.1051870

**Published:** 2022-12-07

**Authors:** Yawen Wang, Yeqing Gu, Jian Huang, Hongmei Wu, Ge Meng, Qing Zhang, Li Liu, Shunming Zhang, Xuena Wang, Juanjuan Zhang, Shaomei Sun, Xing Wang, Ming Zhou, Qiyu Jia, Kun Song, Junsheng Huo, Bing Zhang, Gangqiang Ding, Peng Du, Kaijun Niu

**Affiliations:** ^1^Air Force Medical Center of PLA, Beijing, China; ^2^School of Public Health of Tianjin University of Traditional Chinese Medicine, Tianjin, China; ^3^Nutrition and Radiation Epidemiology Research Center, Institute of Radiation Medicine, Chinese Academy of Medical Sciences & Peking Union Medical College, Tianjin, China; ^4^Chinese Center for Disease Control and Prevention National Institute for Nutrition and Health, Beijing, China; ^5^Nutritional Epidemiology Institute and School of Public Health, Tianjin Medical University, Tianjin, China; ^6^Department of Toxicology and Sanitary Chemistry, School of Public Health, Tianjin Medical University, Tianjin, China; ^7^Health Management Centre, Tianjin Medical University General Hospital, Tianjin, China; ^8^Tianjin Key Laboratory of Environment, Nutrition and Public Health, Tianjin, China; ^9^Center for International Collaborative Research on Environment, Nutrition and Public Health, Tianjin, China

**Keywords:** 25-hydroxyvitamin D, 25-hydroxyvitamin D_2_, 25-hydroxyvitamin D_3_, irisin, sarcopenia

## Abstract

**Background:**

Emerging evidence suggests sarcopenia, which is involved in the serum vitamin D deficiency and development of abnormal muscle metabolism, is predominately centered in the general older population. In the present study, we aimed to explore the relationship between the level of serum vitamin D and irisin concentrations in the older adults with sarcopenia.

**Methods:**

A cross-sectional study was conducted which included 422 sarcopenia participants (146 males and 276 females). Sarcopenia was assessed according to the recommended diagnostic criteria of the Asia Working Group for Sarcopenia (AWGS). The levels of serum 25-hydroxyvitamin D (25(OH)D), 25-hydroxyvitamin D_2_ (25(OH)D_2_) and 25-hydroxyvitamin D_3_ (25(OH)D_3_) were determined by LC-MS/MS. Irisin levels were measured by enzyme-linked immunosorbent assay (ELISA). The relationship between serum concentration of vitamin D and irisin were determined using multiple linear regression analysis.

**Results:**

After adjustment for potential confounding factors, a significant and positive relationship between changes in irisin across 25(OH)D, and 25(OH)D_3_ was observed (standard regression coefficients of 0.150 and 0.151, respectively, *P* < 0.05). However, no significant relationship was observed between serum vitamin D concentrations and irisin levels in males.

**Conclusions:**

This study demonstrated that a higher level of serum vitamin D is independently related to the increment of irisin in sarcopenia females, not in males. These investigations need to be verified in other large-scale prospective studies.

## Background

Sarcopenia is defined as a progressive and generalized skeletal muscle disorder, the incidence of which ranges between 5 and 15% in 65-year-olds, and as high as 50% over 80 years old (1). The risk of falls, fractures, disabilities, hospitalization and mortality increased significantly with the development of sarcopenia (2, 3). An increasing body of evidence suggests that sarcopenia may affect detrimental myokines and hormonal substances metabolic abnormalities, such as irisin, myostatin, interleukin-6 and follistatin (4). Irisin, which is a hormone carried by blood and mainly produced by fibronectin type III domain containing 5 (FNDC5) in muscle tissue. It is a dependent protein of the peroxisome proliferator-activated receptor-γ coactivator-1α (PGC-1α) (5). The expression of PGC-1α and FNDC5 in skeletal muscle are two major gene components of muscle exercise, which is a regulator protein and a precursor of irisin, respectively. In fact, irisin is a potential bridge between muscle and other tissues and organs, primarily responsible for energy metabolism and acts as a positive regulating factor of muscle mass (6). A significant decrease in the irisin level is signed to be a biomarker of muscle weakness and atrophy (7). A cross-sectional study indicated that per 1.0 ng/mL decrease of irisin was associated with an increased prevalence of sarcopenia, with an odds ratio of 1.95 (95% CI: 1.33–2.87) (8). Another study showed that the circulating irisin was significantly lower in the sarcopenia group, and the high irisin was associated with lower odds of sarcopenia in postmenopausal women (9).

The pathophysiology mechanisms of sarcopenia involve the alteration of serum micronutrient status (10). Vitamin D is discovered to be the earliest pro-steroid hormone to arise on earth. It is reported that more than 1 billion individuals are possessing vitamin D insufficiency or deficiency, especially in the older population (11). The deficiency of vitamin D due to sarcopenia is thought to underlie the pathogenesis of low muscle mass, muscle strength, and muscle function (12). Vitamin D thus performs a critical role in the regulation of skeletal muscle metabolic homeostasis. Previous studies have indicated that the level of vitamin D is evaluated as an explanation for the reason of sarcopenia (10, 13, 14).

Many animal studies explored the detailed mechanism of serum vitamin D and irisin on skeletal muscle (15, 16). Compelling evidence has shown that sarcopenia induced lower serum irisin. Moreover, recent studies have demonstrated that several possible risk factors of sarcopenia included irisin and 25(OH)D (9). Therefore, clarifying the common mechanisms of irisin and vitamin D is a crucial step toward providing early prevention and treatment. We speculate that vitamin D and irisin have inherent conjunction during the prevalence and progression of sarcopenia. However, human studies on the relationship between vitamin D status and irisin in the older population with sarcopenia remained unknown. The study aimed to investigate whether serum vitamin D concentrations are related to the level of irisin among older individuals with sarcopenia.

## Methods

### Study participants

The Tianjin Chronic Low-grade Systemic Inflammation and Health (TCLSIH) Cohort Study is a prospective cohort involving >100,000 study participants, a large prospective dynamic cohort study initially designed to investigate the relationships between chronic low-grade systemic inflammation and health status. Additional information on the study design of the TCLSIH has been examined elsewhere (17). At the time of enrollment, participants attended a self-designed structured questionnaire survey including sociodemographic characteristics, diet, lifestyle, household income, occupation, and educational level, etc., and completed comprehensive health examinations. The study procedures were provided by the Institutional Review Board of Tianjin Medical University, and all participants provided written informed consent.

The assessment of sarcopenia was followed the diagnostic approach of the AWGS (18). Participants were recruited as sarcopenia patients according to the following conditions: (1) low muscle mass, the skeletal muscle mass index (SMI) was calculated as follows: SMI (kg/m^2^) = appendicular muscle mass (kilograms) / (height [meter])^2^, which was an effective measure of sarcopenia. The cutoff values of SMI were 7.0 kg/m^2^ in men and 5.7 kg/m^2^ in women by using Bioelectrical Impedance Analysis. (2) low muscle strength. A handgrip strength was measured by a hand-held dynamometer (EH101; CAMRY, Guangdong, China). In the criteria, sarcopenia was defined as grip strength <26 kg for men and <18 kg for women. (3) low physical performance. Gait speed over a distance of 4 meter was measured to evaluate physical performance. Studies have shown that the reference value for low physical capacity was <1 m/s (19). According to the above diagnostic criteria, a total of 463 participants have attended this research. Participants with incomplete data collection on questionnaire and error measurement of irisin and vitamin D values were excluded (*n* = 41). As a result of these exclusions and diagnosis of sarcopenia, 422 participants (females, 65.4%) were included in this cross-sectional study.

### Measurement of vitamin D

Study nurses collected the sarcopenia older participants anthropometric details and blood samples. The fasting blood samples were routinely drawn from participants using venipuncture. Blood samples were protected from light and isolated the serum immediately. Stabilized samples were stored at −80°C. Vitamin D in the serum is in the form of 25(OH)D_2_ and 25(OH)D_3_, which were measured using a liquid chromatography–triple quadrupole mass spectrometry (LC-MS/MS, SCIEX Triple Quad 4500MD). The concentration of 25(OH)D was the sum of 25(OH)D_2_ and 25(OH)D_3_. According to the manufacturer's notes, the coefficient of variance of intra- and inter assay were both <15%. The linear ranges were 2.0–100 ng/mL for 25(OH)D_2_ and 4.0–200 ng/mL for 25(OH)D_3_.

### Measurement of irisin

To assess irisin, blood samples were centrifuged for 5 min at 3000rpm/min, then serum was stored at −80°C. Serum irisin was determined by the principle of competitive enzyme immunoassay according to ELISA kit (Cat. no: EK-067-29, Phoenix Pharmaceuticals Inc., CA, USA). The maximal intra- CV and inter-assay CV were <10 and <15%, respectively, meanwhile, the normal detection range was 5.8–23.2 ng/ ml supported by the manufacturer. The kit was commercially reliable and highly sensitive.

### Measurement of other variables

Height and body weight were measured by using a calibrated balance scale. Body mass index (BMI, kg/m^2^) was calculated as weight divided by height. Body fat composition was assessed by a multifrequency bioelectrical impedance analyzer (In-Body720; Biospace Co, Seoul, Korea). With full consent, age, sex, marital status, educational level, household income, employment status, smoking status, alcohol-consumption status, physical activity, living alone status and the use of nutritional supplement were obtained from the health questionnaire records. Baseline values of individual history of diseases (cardiovascular disease, stroke, cancer, diabetes, hypertension, and hyperlipidemia) were obtained through collecting personal health records and relevant biochemical index evaluation (20). The dietary pattern and total energy intake values were assessed from the food frequency questionnaire (FFQ). Dietary pattern divided into three categories: “sweets,” “healthy,” and “animal foods” dietary pattern. Depressive symptoms were evaluated based on the 20-item of the Chinese version of the Self-Rating Depression Scale (SDS) with cutoff value of 45 (21). A fasting blood sample was drawn and collection data was recorded. For information about the sunlight exposure, it was quantified asking the participant to provide the outdoor activity time in summer/autumn or winter/spring (22).

### Statistical analysis

Statistical analyses of the data were conducted with Analysis System 9.3 edition for Windows (SAS Institute Inc., Gary, NC, USA). Study population characteristics according to sex are reported as means (with 95% confidence interval, CI) for continuous variables and percentages for categorical variables. The concentrations of serum 25[OH]D, 25[OH]D_2_ and 25[OH]D_3_ were used as independent variable, and the level of serum irisin was used as dependent variable separately. The relationship between serum vitamin D and irisin was assessed using multiple linear regression analysis adjusted for age, BMI, physical activity, smoking status, drinking status, history of diseases (cardiovascular disease, stroke, cancer, hypertension, hyperlipidemia, and diabetes), total energy intake, dietary pattern, education level, married status, living alone status, employment status, depressive symptoms and season of average outdoor time, body fat ratio and the use of nutritional supplement. β and standard β values were calculated. All tests were two- tailed and *P* < 0.05 was defined as statistically significant.

## Results

The study sample included 422 sarcopenia subjects aged 69.9 years (range: from 66.1 to 74.1 years), all baseline characteristics are shown in Table 1. There were 146 men and 276 women with complete data for sarcopenia analyses. Multiple linear regression analyses were presented to confirm the crude and adjusted relationship between serum vitamin D concentration and irisin in Table 2. Results of analysis showed a positive relationship between serum 25(OH)D and 25(OH)D_3_ levels and irisin in females using age- and BMI-adjusted model, standard regression coefficient (SRC) = 0.135, *P* < 0.05 and SRC = 0.132, *P* < 0.05 respectively. After adjusting for potential confounders, irisin distinctly showed a significant relationship with 25(OH)D [SRC = 0.150; *P* < 0.05], and 25(OH)D_3_ [SRC = 0.151; *P* < 0.05] in females. However, no significant interactions between 25(OH)D_2_ and irisin were observed in the final models [SRC = 0.032; *P* = 0.606]. Additionally, in males, the crude SRC of irisin across serum vitamin D were presented. Adjusted irisin levels across serum 25(OH)D, 25(OH)D_2_ and 25(OH)D_3_, the values were SRC = −0.073, *P* = 0.423; SRC = −0.096, *P* = 0.304 and SRC = −0.071, *P* = 0.434 respectively. The analysis with multiple linear regressions did not indicate nonlinear relationship between 25(OH)D, 25(OH)D_2_ and 25(OH)D_3_ concentrations and irisin in males.

**Table 1:** Baseline characteristics of participants (*n* = 422).

**Characteristics**	**All**	**Men (34.6%)**	**Women (65.4%)**	***P* value^a^**
Number of participants	422	146	276	
Age (years)	69.9 (66.1, 74.1) ^b^	72.7 (68.0, 76.9)	68.7 (65.8, 72.7)	<0.0001
BMI (kg/m^2^)	23.7 (21.8, 25.8)	23.5 (21.4, 25.0)	24.1 (22.0, 26.0)	0.0551
Body fat ratio (%)	33.6 (27.0, 38.6)	26.7 (22.1, 31.5)	37.0 (31.7 40.9)	<0.0001
Total energy intake (kcal/day)	1,909.5 (1,519.2, 2,385.9)	2,193.1 (1,773.2, 2,677.1)	1,799.0 (1,404.8, 2,191.8)	<0.0001
“Sweets” dietary pattern score	−0.20 (−0.61, 0.48)	0.02 (−0.52, 0.80)	−0.24 (−0.74, 0.25)	0.017
“Healthy” dietary pattern score	−0.30 (−0.51, 0.24)	−0.34 (−0.54, 0.20)	−0.28 (−0.51, 0.27)	0.52
“Animal foods” dietary pattern score	−0.40 (−0.73, 0.17)	−0.04 (−0.66, 0.78)	−0.46 (−0.75, −0.13)	<0.0001
SDS score^c^	36.0 (29.0, 41.0)	35.0 (28.0, 41.0)	36.0 (29.0, 41.0)	0.18
Smoking status (%)				<0.0001
Current smoker	36.65^d^	48.25	30.48	
Ex-smoker	7.77	14.69	4.09	
Non-smoker	55.58	37.06	65.43	
Alcohol drinking status (%)				<0.0001
Everyday	4.05	16.67	0.00	
Sometime	6.76	22.22	1.79	
Ex-drinker	4.05	16.67	0.00	
Non-drinker	85.14	44.44	98.21	
PA (≥23 MET × hour/week, %)	33.18	40.41	29.53	0.055
Education level (≥middle school, %)	14.37	22.95	9.27	<0.001
Marital status (married, %)	99.14	97.62	100.0	0.021
Living alone (yes, %)	13.12	12.90	13.24	0.93
History of disease (%)				
Hypertension	50.71	50.68	50.72	0.53
Hyperlipidemia	46.68	34.25	53.26	<0.0001
Stroke	5.69	6.16	5.43	<0.0001
Cancer	0.24	0.00	0.36	<0.0001
Diabetes	13.74	13.0	14.13	<0.0001
Season of average time outdoors				
Summer (≥3 h/d, %)	59.94	77.69	50.00	<0.0001
Winner (≥3 h/d, %)	59.05	76.86	49.07	<0.0001
Use of dietary supplements (yes, %)	0.60	0.84	0.46	0.67

BMI, body mass index; PA, physical activity.

a Wilcoxon rank-sum test or chi-square test.

b Continuous variables are expressed as medians (interquartile range) (all such values).

c SDS score were evaluated based on the 20-item of the Chinese version of the Self-Rating Depression Scale.

d Categorical variables are expressed as percentages (all such values).

**Table 2:** Adjusted relationships of serum vitamin D levels and the circulating irisin in sarcopenia (*n* = 422).

	**25-hydroxyvitamin D (ng/mL)**					**25-hydroxyvitamin D**_**2**_ **(ng/mL)**					**25-hydroxyvitamin D**_**3**_ **(ng/mL)**				
**Irisin concentration (ng/mL)**	**β**	**Standard-ized β**	**Standard Error**	**t Value**	***P* Value ^a^**	**β**	**Standard-ized β**	**Standard Error**	**t Value**	***P* Value ^a^**	**β**	**Standard-ized β**	**Standard Error**	**t Value**	***P* Value ^a^**
Male (*N* = 146)															
Model 1^b^	−0.101	−0.052	0.161	−0.63	0.531	−0.071	−0.089	0.067	−1.07	0.285	−0.098	−0.052	0.158	−0.62	0.534
Model 2^c^	−0.106	−0.055	0.161	−0.66	0.510	−0.084	−0.105	0.067	−1.26	0.209	−0.102	−0.054	0.158	−0.65	0.518
Model 3^d^	−0.141	−0.073	0.175	−0.80	0.423	−0.077	−0.096	0.074	−1.03	0.304	−0.134	−0.071	0.170	−0.78	0.434
Female (*N* = 276)															
Model 1^b^	0.163	0.134	0.073	2.23	<0.05	0.039	0.068	0.034	1.13	0.260	0.156	0.131	0.072	2.18	<0.05
Model 2^c^	0.165	0.135	0.073	2.26	<0.05	0.037	0.065	0.034	1.07	0.283	0.158	0.132	0.071	2.22	<0.05
Model 3^d^	0.183	0.150	0.075	2.46	<0.05	0.018	0.032	0.035	0.52	0.606	0.180	0.151	0.073	2.46	<0.05

a Obtained by using multiple linear regression analysis.

b Unadjusted model.

c Adjusted for age, BMI.

d Adjusted for age, BMI, physical activity, smoking status, alcohol-consumption status, individual history of diseases (cardiovascular disease, stroke, cancer, hypertension, hyperlipidemia, and diabetes), total energy intake, dietary pattern score, education level, married status, employment status, SDS score, living alone status, season of average outdoor time, body fat ratio, the use of dietary supplements.

## Discussion

Previous studies not only have shown the relationship between sarcopenia and 25(OH)D (23, 24), but also proved that irisin showed potentially far-reaching effects on muscle function (25, 26). In the present study, we firstly investigate the relationship between serum 25(OH)D status and the circulating levels of irisin in a sarcopenia population. Our results showed that the changes in irisin have a strongly analogous relationship with serum 25(OH)D and 25(OH)D_3_ levels in females, but not in males, implicated that serum are rich in 25(OH)D and 25(OH)D_3_, which may inhibit lower irisin levels induced by sarcopenia in older adults.

In the current study, we adjusted multiple recognized confounding factors. First, we calculated an unadjusted regression model to identify any differences in irisin according to types of serum 25(OH)D concentration. Second, several studies have revealed that irisin is related to age and BMI (27, 28), therefore, we adjusted for these two variables. After adjustment, there is a statistical significantly relationship between 25(OH)D and 25(OH)D_3_ and the level of irisin was observed. Finally, sociodemographic variables, lifestyle factors, medical history, total energy intake and seasonal differences of outdoor time are closely associated with sarcopenia (17). Therefore, we further adjusted for smoking status, drinking status, educational level, occupation, married status, living alone status, family history of the disease, individual history of the disease, total energy intake and outdoor time. After all adjustments were made, the standard regression coefficient of female sarcopenia participants with irisin were 0.143 and 0.145 in interaction with 25(OH)D and 25(OH)D_3_. However, males did not show any significant difference. The gender-specific difference between the concentration of irisin and vitamin D in this study seem to be explained by the innately higher amount of subcutaneous fat mass in women, which may stimulate a higher biological activity and lead to a higher irisin concentration (29). At the same time, subcutaneous adipose tissue is closely related to estrogen, which may absorb more vitamin D molecules produced from the skin because of its fat-soluble properties (30). These results indicate that the associated changes in serum vitamin D and irisin are more affected by estrogen levels and body fat content. Studies are needed to confirm this hypothesis.

Sarcopenia is a hallmark of the aging process. The occurrence of sarcopenia causes abnormality in many physiological and biochemical indicators (1, 13, 25). A meta-analysis reported by Olivier showed that vitamin D supplements increased muscle strength in people who had 25(OH)D levels <30 nmol/L (approximately 12 ng/mL), and seemed more valid in older people over 65 years (31), which suggested that in the sarcopenia older population with lower levels of serum 25(OH)D, vitamin D performed a small but significant positive effect. A previous study showed that the cut-off value of serum irisin level of 8.46 ng/mL performed maximal sensitivity (68%) and specificity (69%), the relationship between irisin and sarcopenia was not limited by the adjustment of BMI and age (8), indicated that irisin is a novel and independent predictor for sarcopenia. Previous studies demonstrated that irisin was a stronger determinant of bone mineral status (32), increasing bone formation and decreasing bone resorption, leading to reduced risk of osteoporosis. Irisin had been shown to play an important role in promoting osteoblastogenesis and reducing osteoclastogenesis (33). Therefore, we speculate the beneficial effects of irisin on bone protection in postmenopausal women with sarcopenia. Although there are several studies underlined the impact of sarcopenia on serum 25(OH)D and irisin respectively, few studies have evaluated the biochemical parameter of cross interaction in older sarcopenia participants. These results imply that earlier vitamin D supplementation is needed to preserve irisin at a higher level, to delay the development of sarcopenia in the older population.

Previous animal studies have probed that lower serum 25(OH)D reduced the energy homeostasis and irisin levels (34). Serum 25(OH)D concentrations affect the expression of the parathyroid hormone (S-PTH), which might lead to an activation of PGC1α and result in high irisin secretion into the blood (35, 36). Of note, there is no statistical relationship between 25(OH)D_2_ and irisin in females. Romagnoli et al. (37) found that serum 25(OH)D_2_ seems to be less efficient than 25(OH)D_3_ in decreasing S-PTH levels. We speculated that the changes of irisin in patients with sarcopenia may have an impact on the inverse relationship between S-PTH concentrations and 25(OH)D_3_. The changes in serum 25(OH)D_2_ was not significant and will require further investigation. Moreover, vitamin D receptor expression affects the functional response of muscle cells to 25(OH)D, which is expressed in human skeletal muscle. VDR expression is down-regulated with age and sarcopenia (38). Further studies will be clarified the exact mechanisms of 25(OH)D mediating the effect of irisin on older sarcopenia participants.

The major strength of this study is that we first report the relationship between serum vitamin D and concentrations of irisin with sarcopenia in the general population. Moreover, multiple potential confounders were considered in this study, such as sociodemographic factors, health status, dietary patterns and seasonal influences. This study has several limitations. First, having only included older sarcopenia participants in cross-sectional design, causality could not be provided. Second, participants were recruited from a single area and the included sample was relatively small, which might have negatively affected the power of data analysis. In addition, some hormonal levels might have been overlooked due to the limited on sample collection. Although these may influence the metabolic levels the irisin and vitamin D in sarcopenia (39), it indicated that the analysis of data were relatively stable over time according to our published studies. Besides, we could not investigate familial and genetic details prior to the study, though irisin levels can be maternally inherited. More research is necessary to detect the relationship between these observations in terms of genetic inheritance (40).

## Conclusions

In conclusion, this study revealed that 25(OH)D is positively related to the concentration of irisin in sarcopenia females. The prevalence of sarcopenia increased stepwise because of the risk factors, further prospective epidemiologic studies with to replicate our findings and investigate the underlying mechanisms that will be done in the future. Therefore, further studies with large sarcopenia participants needed to confirm our results.

## Data availability statement

The original contributions presented in the study are included in the article/supplementary material, further inquiries can be directed to the corresponding authors.

## Ethics statement

The studies involving human participants were reviewed and approved by the Medical Ethics Committee of the Tianjin Medical University with the reference number of TMUhMEC 201430. The patients/participants provided their written informed consent to participate in this study.

## Author contributions

YW and YG analyzed data and wrote the paper. JH, HW, GM, QZ, LL, SZ, XuW, JZ, SS, XiW, MZ, QJ, KS, JH, BZ, and GD conducted data collection or management. PD and KN designed the research and had primary responsibility for the final content. All authors contributed to the article and approved the submitted version.
